# Spontaneous Intracranial Hypotension: A Rare Etiology of Subarachnoid Hemorrhage

**DOI:** 10.7759/cureus.94381

**Published:** 2025-10-12

**Authors:** Maria Eduarda Moniz, Elisa Caldeira, João Loja, Carolina Olim, Hugo Dória

**Affiliations:** 1 Internal Medicine, Serviço de Saúde da Região Autónoma da Madeira, Entidade Pública Empresarial da Região Autónoma da Madeira (SESARAM, EPERAM), Funchal, PRT; 2 Radiology, Serviço de Saúde da Região Autónoma da Madeira, Entidade Pública Empresarial da Região Autónoma da Madeira (SESARAM, EPERAM), Funchal, PRT

**Keywords:** headache, non-aneurysmal subarachnoid hemorrhage, non-traumatic subarachnoid hemorrhage, spontaneous intracranial hypotension, stroke

## Abstract

Subarachnoid hemorrhage (SAH) is a rare complication of spontaneous intracranial hypotension (SIH), and the underlying pathophysiological mechanism linking these two conditions is not yet fully understood.

We report the case of a previously healthy 49-year-old female patient who presented with a bilateral fronto-occipital headache that worsened over the course of five days, accompanied by neck pain, nausea, photophobia, and phonophobia. The headache was refractory to analgesic treatment and lessened with recumbency. A brain CT scan revealed a mild SAH on the right Sylvian fissure, while CT angiography (CTA) and digital subtraction angiography (DSA) were unremarkable. MRI demonstrated features consistent with SIH. In this patient, MRI findings supported the hypothesis of brain sag with bridging vein strain as the underlying mechanism. An epidural blood patch was considered but deferred due to spontaneous clinical improvement without recurrence.

This case report emphasizes that SIH should be considered in patients presenting with non-aneurysmal SAH, particularly if the headache has orthostatic features.

## Introduction

Spontaneous intracranial hypotension (SIH) results from disturbances in the production, flow, and absorption of cerebrospinal fluid (CSF) [1]. Classically, it presents with an orthostatic headache and requires a high level of clinical suspicion, as it is often underdiagnosed. MRI is crucial for the diagnosis. Rarely, it can be complicated by cerebral venous thrombosis, subdural hematoma, and subarachnoid hemorrhage (SAH), particularly if there is a delay in diagnosis [2,3]. SAH is a life-threatening condition, and its correlation with SIH has been the subject of investigation. In cases where vascular imaging, such as CT angiography or digital subtraction angiography, reveals no vascular abnormality, the hemorrhage is classified as angiography-negative SAH.

We report the case of a patient who presented with SAH caused by SIH. This case is notable for a possibly shorter interval between the onset of SIH and SAH symptoms, a distinctive combination of MRI findings, and successful conservative management without targeted leak therapy.

## Case presentation

A previously healthy 49-year-old woman presented to the emergency department (ED) with a worsening bilateral fronto-occipital headache, accompanied by neck pain, nausea, photophobia, and phonophobia over the course of five days. The headache improved with recumbency but acutely worsened on the fifth day. On that day, she continued to experience the same symptoms but reported a sudden and marked increase in pain intensity. She had already visited the ED twice (once the day before and once on the morning of presentation), complaining of a headache refractory to analgesic treatment. She denied any history of fever, chills, visual disturbances, weakness, numbness, or prior trauma. Additionally, she reported no use of tobacco, alcohol, or illicit drugs. Vital signs were unremarkable. The Glasgow Coma Scale (GCS) score was 15. Percussion of the paranasal sinuses elicited no tenderness. Palpation of the cervical region did not exacerbate the neck pain. Pupils were symmetric, isocoric, and reactive to light. The neurological examination revealed no deficits in strength, sensation, coordination, or gait. Meningeal signs were not present. Laboratory tests and an electrocardiogram obtained in the ED were unremarkable.

**Figure 1:**
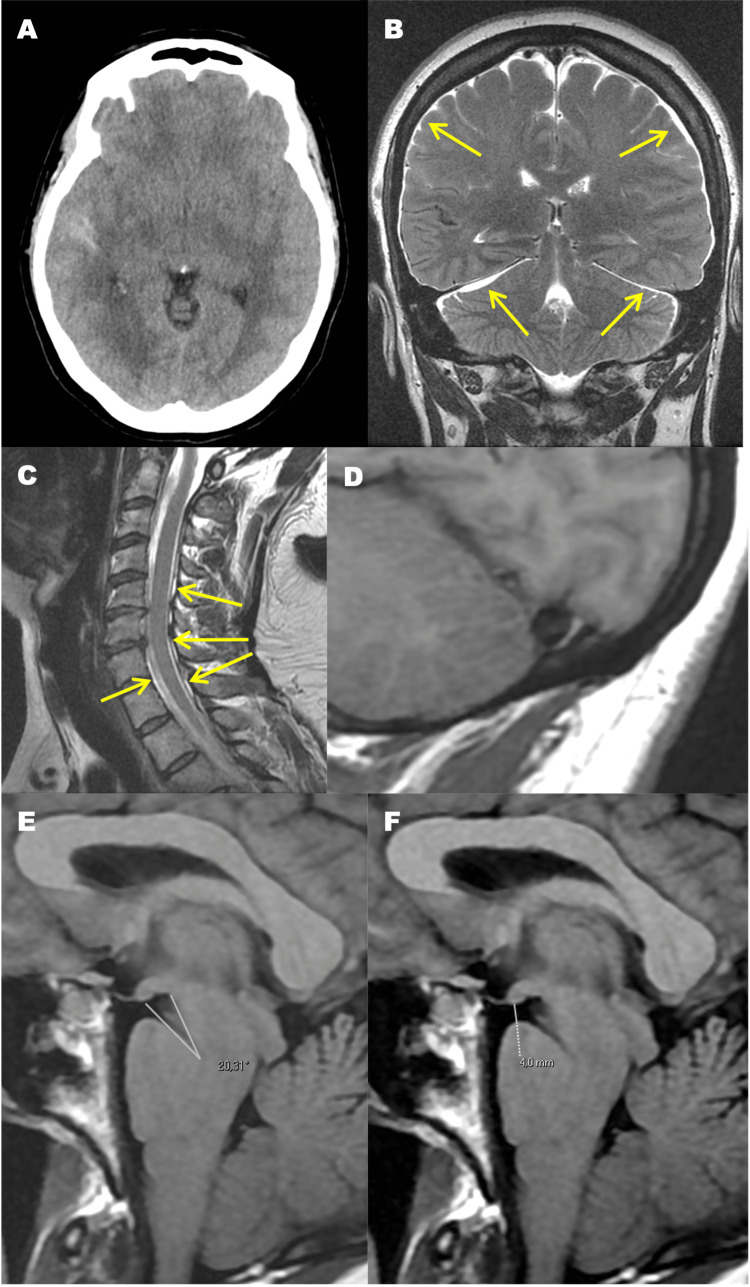
Imaging findings (A) CT brain scan, axial slice, showing subarachnoid hemorrhage on the right Sylvian fissure. (B) Brain MRI, coronal T2-weighted image, demonstrating subdural effusions (arrows). (C) Cervical MRI, sagittal T2-weighted image, showing spinal epidural collections (arrows). (D-F) Brain MRI, sagittal T1-weighted images, showing venous distension (D), a narrow pontomesencephalic angle (E), and a short mamillopontine distance (F).

A non-contrast CT scan on admission showed SAH on the right Sylvian fissure (Figure 1A). CTA performed on admission and DSA two weeks later revealed no vascular abnormalities, including aneurysms or arteriovenous shunts. A brain and cervical MRI performed two weeks later showed subdural effusions, spinal epidural collections, venous distension, as well as a narrow pontomesencephalic angle and a short mamillopontine distance (Figure 1B-1F). The distribution of subarachnoid blood was not consistent with a perimesencephalic SAH pattern. Lumbar puncture was not performed, but the diagnosis of SIH was supported by the characteristic MRI findings. The patient was admitted to the Stroke Unit for monitoring and etiological investigation.

She was treated with nimodipine, intravenous saline for hydration, non-opioid analgesics (acetaminophen and non-steroidal anti-inflammatory drugs); bed rest was also advised. The headache was effectively controlled with analgesics. Nimodipine was initiated empirically, following standard subarachnoid hemorrhage protocols to reduce the risk of delayed cerebral ischemia, although the hemorrhage was non-aneurysmal. Treatment was continued for 21 days. A vasospasm screening protocol with serial transcranial Doppler ultrasonography was performed during hospitalization, and no evidence of vasospasm was detected. Although MRI findings were suggestive of SIH, an epidural blood patch or targeted leak therapy was not pursued due to spontaneous clinical improvement and sustained resolution of symptoms. During hospitalization, she remained hemodynamically stable, normocardic, afebrile, and without neurological deficits. As clinical improvement was noted, the patient remained under surveillance, and no further treatment was required. The patient was reassessed in the outpatient clinic, and the headache did not recur. At discharge, the patient was advised to avoid strenuous physical activity, maintain adequate hydration, and promptly return for evaluation in case of recurrent headache or neurological symptoms. She was also informed about the possibility of recurrence and the need for follow-up.

## Discussion

SAH is a medical emergency that can be caused by traumatic or non-traumatic etiologies, with approximately 85% of non-traumatic cases resulting from a ruptured aneurysm. It is associated with a high mortality rate, regardless of its etiology [4]. SIH has been described as a rare etiology of non-aneurysmal SAH [2].

The correlation between SAH and SIH is not fully understood [3]. The Monro-Kellie theory states that the sum of the volumes of the brain, CSF, and intracranial blood is constant. Therefore, in the presence of intracranial hypotension, abnormalities such as meningeal enhancement, subdural fluid collections, enlargement of the pituitary gland, engorgement of cerebral venous sinuses, and spinal epidural venous plexus can be seen on brain MRI scans [3,5]. Some authors propose that engorgement and stretching of intracranial veins (possibly with dural rupture) and/or the brain sagging itself may result in the rupture of cortical veins or venous plexuses [3,6].

In 2023, Cao et al. reported two cases of SAH caused by SIH in China. In their article, the authors also reviewed the literature and identified eight additional cases [3].

Regarding clinical presentation, in some cases, SIH can mimic SAH [7]. Patients with SAH typically present with a severe, sudden-onset (thunderclap) headache, frequently associated with nausea, vomiting, cervical pain, and photophobia [4]. Patients with SIH can also develop a thunderclap headache in approximately 14% of cases, but more commonly, SIH is characterized by a gradual-onset orthostatic headache that relieves with recumbency [1,7,8].

In this case, the gradual-onset headache with orthostatic features initially experienced by the patient supports the hypothesis that SIH preceded the SAH. Furthermore, the headache became more intense on the fifth day, which may suggest that it was then that the SAH took place. We speculate that the interval between the symptoms of SIH and the symptoms of SAH was approximately five days, which is shorter than that reported in any of the previously mentioned cases of the 2021 review [3]. However, this interpretation remains hypothetical and should be considered in light of important limitations, including the absence of a documented leak site, the lack of CSF opening pressure measurement, and the reliance on single-time-point imaging. In addition, our case presents a unique combination of imaging findings and a management approach that did not include targeted leak therapy.

Our patient experienced a progressively worsening headache, refractory to medication, which should be considered a red flag when ruling out secondary headache. Additionally, the headache worsened in the upright position and improved when lying down, another telltale sign of secondary headache. Despite these warning signs, imaging studies were delayed.

## Conclusions

Although the most common cause of non-traumatic SAH is aneurysmal rupture, other potential etiologies should be considered when imaging studies exclude vascular abnormalities. Thus, SIH should be considered in patients presenting with non-aneurysmal SAH. This is particularly important when orthostatic features accompany the headache.
